# Concentric macular rings and OCT corrugations in foveal hypoplasia: proof of concept for an optical interference mechanism

**DOI:** 10.1186/s40942-025-00755-5

**Published:** 2025-12-29

**Authors:** Ari H. August, Ralph C. Eagle, Tatyana Milman, Rebecca Procopio, Bruce M. Schnall, José S. Pulido

**Affiliations:** 1 Department of Ophthalmology, Sidney Kimmel Medical College of Thomas Jefferson University, 1025 Walnut St #100, Philadelphia, PA 19107 USA; 2 Wills Eye Hospital, 840 Walnut Street, Philadelphia, PA 19107 USA

**Keywords:** Foveal hypoplasia, Oculocutaneous albinism, Fingerprinting, Macular ripples, Ophthalmic imaging, OCT corrugations, Imaging artifact, Interference, Newton’s rings, Retinal imaging, Optical coherence tomography, Scanning laser ophthalmoscopy

## Abstract

**Background:**

Concentric macular rings (CMR) and Henle fiber layer (HFL) corrugations, potential clinical biomarkers of foveal hypoplasia, have been observed in laser fundus photographs and optical coherence tomography (OCT) images, respectively. Some believe these findings represent true anatomical structural changes while others hypothesize that they are artifacts of interference; however, to our knowledge, no previous study has provided evidence to support either theory.

**Methods:**

This retrospective case series analyzed CMR and OCT corrugations in 3 patients (6 eyes) with foveal hypoplasia. Dark fringe diameters of CMR at different wavelengths (λ: red = 635, green = 532, blue = 488 nm) and OCT corrugations of the first five dark fringes were measured. Dark fringe diameter was compared to fringe order (n) and λ using linear regression and t-tests.

**Results:**

CMR fringe measurements demonstrated linear relationships between radius^2^ (r^2^) and fringe order at all wavelengths (mean R^2^: 0.949, SD: 0.022, range: 0.911–0.985). OCT corrugation measurements also exhibited strong linearity with fringe order (R^2^ = 0.998, 0.946). Fringe r^2^ increased with wavelength but exceeded theoretical predictions (*p* < 0.05), especially among ratios including shorter λ (mean difference r^2^ red:green = 0.104, green:blue = 0.292, red:blue = 0.468).

**Conclusions:**

This study is the first to provide evidence supporting an etiologic mechanism of CMR and HFL corrugations, showing correlation with physical phenomena of interference. CMR and HFL corrugations correlate with fringe order (n) and λ in a manner consistent with Newton’s rings (r^2^ = nR’λ, *r* = radius, R’ = radius of curvature), suggesting these phenomena are imaging artifacts. Further research validating these results will be important.

**Trial registration:**

This study does not report the results of a health care intervention on human participants.

**Clinical trial disclosure:**

This study does not report results of a clinical trial.

## Background

Foveal hypoplasia (FH) is a spectrum of foveal pit and foveal avascular zone (FAZ) underdevelopment [1]. FH has been associated with anomalous imaging phenomena of “concentric macular rings” (CMR), a pattern of successive annular fringes observed with specific en face retinal imaging modalities [2–6]. FH has also been associated with “corrugations,” alternating hyper- and hyporeflective bands, in the Henle fiber layer (HFL) on optical coherence tomography (OCT), thought to be a cross-sectional correlate of CMR [4]. Proposed mechanisms for CMR and HFL corrugations fall into two categories: those suggesting true intrinsic structural abnormalities (e.g., Henle fiber orientation and distribution, cystoid spaces) and those which postulate these patterns are extrinsic optical artifacts (e.g., optical interference, phase retardation); however, all have been conjecture [2, 4–8].

In this pilot study, we employ novel quantitative methods to evaluate whether the morphology of CMR and HFL corrugations adhere to physical laws of optical interference, as predicted by wave optics. Demonstration of such conformity would support that these phenomena represent extrinsic imaging artifacts.

## Methods

### Study design and participants

**Fig. 1:**
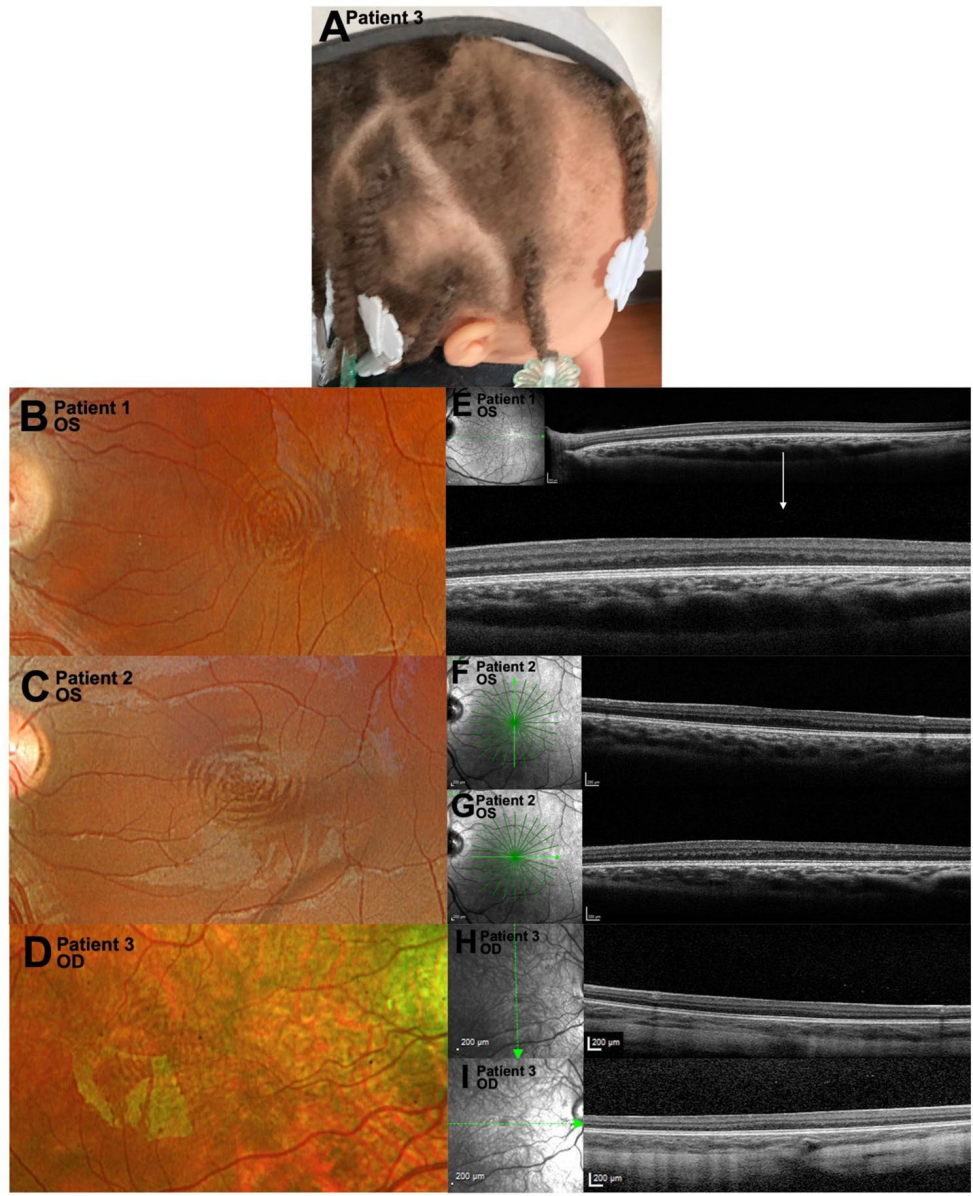
Oculocutaneous albinism: clinical and imaging findings. **A**. External photograph of patient 3, demonstrating hypopigmentation of hair and skin similar to that observed in patients 1 and 2. **B**-**D**. Concentric macular rings (CMR) visible on RGB (**B**-**C**) and RG (**D**) color confocal scanning laser ophthalmoscopy (cSLO). **E**-**I**. Horizontal (**E**,**G**,**I**) and vertical (**F**, **H**) optical coherence tomography (OCT) sections demonstrating foveal hypoplasia and alternating hyperreflective and hyporeflective bands radiating out from presumed fovea; corresponding near-infrared reflectance images do not show prominent CMR

This was a retrospective observational case series of three patients (six eyes) with clinically confirmed foveal hypoplasia who demonstrated visible CMR on confocal scanning laser ophthalmoscopy (cSLO) (Fig. 1) [9, 10]. Patients were identified from the ophthalmic genetics clinic at Wills Eye Hospital based on clinical diagnosis and availability of high-quality multimodal imaging. This was a purposive sample selected to explore optical interference patterns, rather than a systematic or consecutive cohort. All three patients were found to have genetically confirmed oculocutaneous albinism type 2 (OCA2; OMIM phenotype ID #203200); this was coincidental and not a selection criterion. The study was approved by the institutional review board of Wills Eye Hospital, with a waiver of informed consent. All research adhered to the tenets of the Declaration of Helsinki.

### Imaging acquisition

**Fig. 2:**
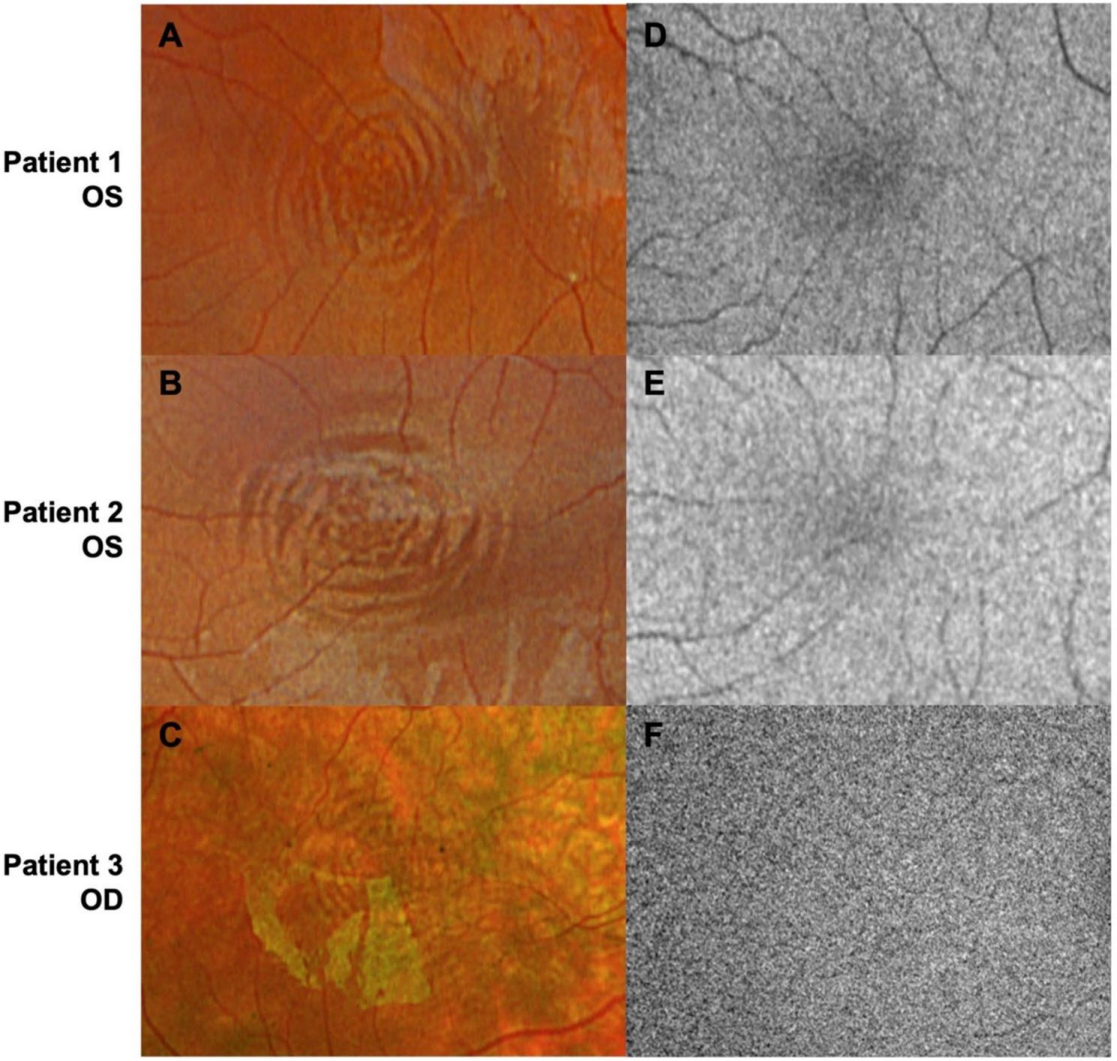
Oculocutaneous albinism: scanning laser ophthalmoscopy (cSLO) images. **A**-**C**. RBG (**A**–**B**) and RG (**C**) Color cSLO fundus photography with CMR. **D**–**F**. Fundus autofluorescence corresponding to color cSLO fundus photograph shown. CMR may be faintly visible when compared to their respective color images

Color fundus imaging was performed using a confocal scanning laser ophthalmoscope (Optos California, P200DTx; Optos Inc., Marlborough, MA, USA). Two patients were imaged with the full three-color configuration (RGB cSLO; 635 nm [red], 532 nm [green], and 488 nm [blue]), and one patient with a two-color configuration (RG cSLO; 635 nm and 532 nm) (Fig. 1) [11, 12]. Fundus autofluorescence (FAF) imaging was obtained with the same system using 532 nm excitation (Fig. 2). cSLO-based near-infrared reflectance (NIRR; 815 nm diode laser) was performed (Spectralis, Heidelberg Engineering, Heidelberg, Germany); spectral-domain optical coherence tomography (OCT) was performed on the same platform, which uses a broadband light source spanning 790–910 nm (centered at 850 nm) to form B-scans from interference signals between the sample and reference arms (Fig. 1) [13, 14]. OCT imaging was performed in both horizontal and vertical single-line B-scan modes centered on the fovea, as well as radial scan protocols (12 lines) when possible. All scans were acquired in high-resolution mode with automatic real-time averaging depending on fixation stability (9–25 frames).

### Imaging analysis

**Fig. 3:**
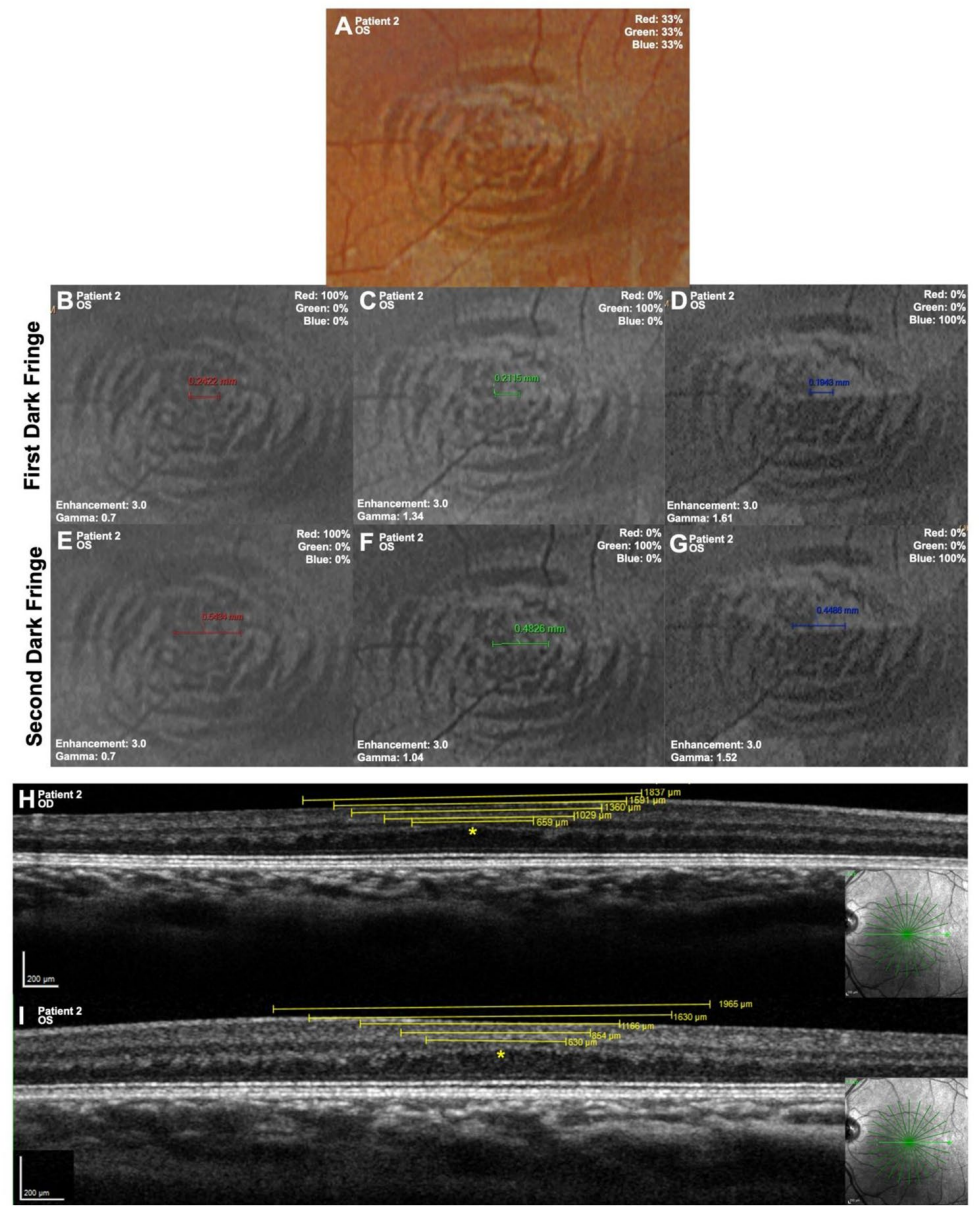
Measurement of concentric macular ring (CMR) dark fringes in patient 2. **A**. cSLO fundus photograph OS, 33% tricolor blend (λ red = 635 nm, λ green = 532 nm, λ blue = 488 nm). **B**–**D**. cSLO fundus photograph OS with first dark fringe diameter measurements (mm) at each wavelength: 100% red (**B**), green (**C**), and blue (**D**). **E**–**G**. cSLO fundus photograph OS with second dark fringe diameter measurements (mm) at each wavelength: 100% red (**E**), green (**F**), and blue (**G**).**H**–**I**. Horizontal OCT sections od (**H**) and OS (**I**) with the first five diameter measurements (µm) of dark fringes radiating out from central fovea (yellow asterisks)

RGB and RG images were analyzed using Optos Advanced software (Optos Inc., Marlborough, MA, USA). For each eye with foveal hypoplasia, the color blend tool was used to isolate each available color channel for separate CMR analysis. Using the software’s annotation feature, a single grader measured the horizontal diameter of the first five discernible dark fringes at each wavelength, from center to center of each dark fringe (Fig. 3A–G).

OCT images were analyzed using HEYEX 2 software (Heidelberg Engineering, Heidelberg, Germany). Horizontal B–scans with clear foveal localization were selected for analysis. OCT imaging of Patients 1 and 3 was limited secondary to nystagmus and age; therefore, only Patient 2 had radial OCT sections available to confirm foveal location and allow more detailed analysis. The foveal center and each dark band within the outer plexiform layer (OPL) were visually identified and the distance between adjacent dark bands was measured with the HEYEX 2 caliper tool (1:1 µm scaling). Measurements were obtained for the first five dark fringes radiating from the foveal center in each eye (Fig. 3H–I). 

All cSLO fringe and OCT band measurements were repeated two weeks apart by the same grader, blinded to initial results. Variability stemmed from fringe order or band identification and was resolved by adjusting gamma or enhancement (cSLO) and contrast (OCT) parameters or selecting clearer frames from the same session to optimize visibility of concentric dark fringes.

### Quantitative and statistical analysis

We hypothesized that CMR follow similar spatial patterning laws analogous to Newton’s rings of optical interference, described by the equation:

$${r^2} = nR{^\prime}\lambda $$

where *r* is fringe radius, *n* is fringe order, *R’* is the radius of curvature (constant), and *λ* is the wavelength of light [15]. The first five dark fringes correspond to *n* = ½, 1½, 2½, 3½, and 4½, respectively.

Data were organized and analyzed in Microsoft Excel (version 16.91; Microsoft Corp., Redmond, WA) and IBM SPSS Statistics (version 29.0.2.0; IBM Corp., Armonk, NY). Linear regression was used to evaluate relationships between dark fringe radius^2^ and fringe order. One-sample and paired t-tests were applied for comparison with theoretical values, with statistical significance defined as *p* < 0.05 (two-tailed). Linear regression was performed comparing squared fringe radius (*r*^*2*^, dependent) to fringe order (*n*, independent) for each wavelength. Experimental and theoretical ratios of squared fringe radius (*r*^*2*^) across wavelengths (red, green, blue) were also compared using one-sample and paired *t*-tests.

cSLO measurment error for *r*^*2*^ was estimated from the Optomap plus resolution (14 µm; absolute error = 1.96 × 10^−10^ m^2^), and determined to be negligible [12]. OCT measurement error for r^2^ of was calculated to account for both the 60 nm range of wavelengths centered around 850 nm (relative uncertainty = 0.0706*r^2^) and the 5 µm lateral resolution of the OCT machine, which contributed to a very small magnitude of error (absolute uncertainty = 25 × 10^−10^ m^2^) [16]. As dark fringe order is exact, uncertainty calculations were not indicated.

## Results

### Patient characteristics

Demographic and clinical features are summarized in Table 1. All patients presented with congenital nystagmus, reduced visual acuity, and cutaneous and iris hypopigmentation (Fig. 1A). Patients 1 and 2 were siblings; Patient 3 was unrelated. All eyes demonstrated bilateral grade 3 foveal hypoplasia (Fig. 1E–I). Each patient carried two pathogenic *OCA2* variants [15, 17–21].

**Table 1:** Demographics and clinical presentation of patients with foveal hypoplasia (FH)

	Patient 1 (P1)		Patient 2 (P2)		Patient 3 (P3)	
Age (y), Sex	11, F		16, M		2.5, F	
MH	None		None		Global developmental delay, G6PD Deficiency	
Parent Ethnicity	**Mother**	**Father**	**Mother**	**Father**	**Mother**	**Father**
	A + EU^a^	A^a^	A + EU^a^	A^a^	AA^a^	AA + PR^a^
Skin Color	Light Brown^b^		Light Brown^b^		Light Brown^b^	
Hair Color	Light Brown^b^		Light Brown^b^		Light Brown^b^	
Iris Color	Light Brown^b^		Light Brown^b^		Light Brown^b^	
Nystagmus	Marked, horizontal		Marked, horizontal		Intermittent, horizontal	
	**OD**	**OS**	**OD**	**OS**	**OD**	**OS**
BCVA	20/100+1	20/200	20/30–1	20/40+2	FF	FF
SPH (D)	+2.50	+2.75	+2.00	+2.00	−8.50	−8.50
CYL (D)	+2.25	+1.75	+2.50	+2.50	+3.50	+3.50
IOP (mmHg)	14	15	16	14	8	7
TID	None	None	None	None	None	None
Other Findings	None	None	Anteriorly displaced Schwalbe lines		None	None
FH [10, 11]	Grade 3	Grade 3	Grade 3	Grade 3	Grade 3	Grade 3
Diagnosis	FH, OCA^c^		FH, OCA^c^		FH, OCA^d^	

A = African; AA = African American; BCVA = best corrected visual acuity; CYL = cylinder; D = diopters; EU = European; FF = fixes on and follows light; FH = foveal hypoplasia; G6PD = glucose-6-phosphate dehydrogenase; IOP = intraocular pressure; MH = medical history; OCA = oculocutaneous albinism; OD = right eye; OS = left eye; PR = Puerto Rican; SPH = sphere; TID = transillumination defects

^a^ no known consanguinity

^b^ lighter than either parent and unaffected siblings

^c^ Invitae Oculocutaneous Albinism panel (Next Generation Sequencing, 23 genes) identified heterozygous pathogenic variants in *OCA2* (OMIM #611409): NM_000275.3:c.819_822delinsGGTC, VCV000198707.25, rs797044784 and NM_000275.3:c.593C > T (p.Pro198Leu), VCV000198063.44, rs183487020; both have been previously associated with the phenotype of OCA.[22]

^d^ GeneDx Nystagmus Xpanded panel (Next Generation Sequencing, 890 genes) identified heterozygous pathogenic variants in *OCA2*: NM_000275.2:c.647_807del, VCV000000952.4, rs1555375711 (not present in the mother) and NM_000275.3:c.1327 G > A, VCV000000955.87, rs121918166 (present in the mother); both have been previously associated with the phenotype of OCA [23–25]

### Concentric macular rings on fundus photography

All patients exhibited CMR on RGB or RG cSLO imaging in both eyes (OU; Fig. 1 B–D). When comparing NIRR and FAF to respective RGB or RG images, CMR visibility was ambiguous; therefore, these modalities were not included in quantitative analyses (Figs. 1, 2).

**Fig. 4:**
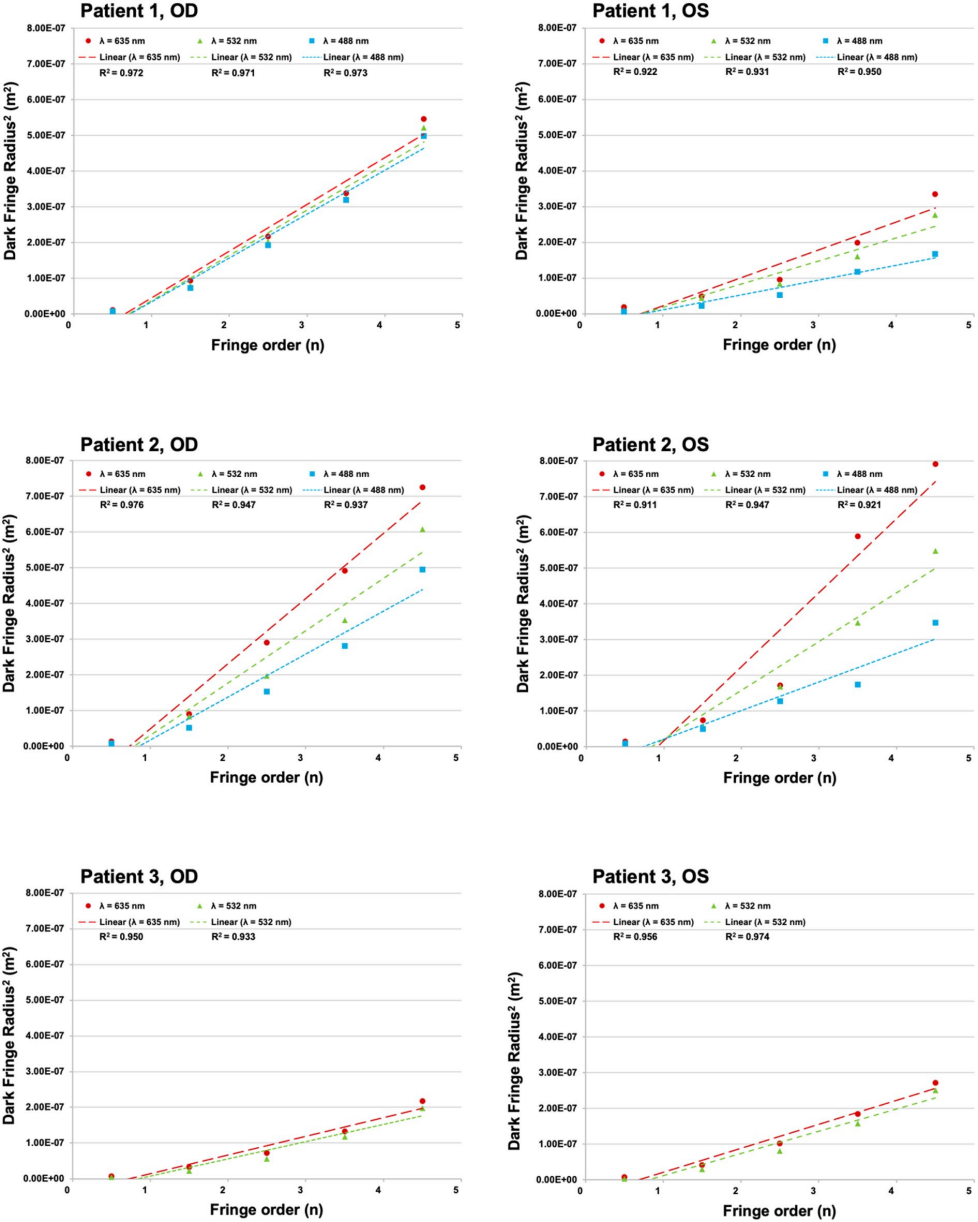
Fundus photography concentric macular ring (CMR) dark fringe radius^2^ (m^2^) vs fringe order (**n**). Photographs taken by confocal scanning laser ophthalmoscopy (optos California). Linear curve curve fit to CMR radius^2^ measurements, *n* = 5, at each available wavelength (λ): λ red = 635 nm, λ green = 532 nm, λ blue = 488 nm. Linear regression was performed for each eye at each λ, with R^2^ values ranging from 0.911 to 0.985

Across 16 linear regression analyses (five fringe orders per wavelength), coefficients of determination (R^2^) were high (mean 0.949, SD 0.022), with all models statistically significant (*p* < 0.05) (Fig. 4, Tables 2, 3). These results indicate a strong linear relationship between squared fringe radius (r^2^) and fringe order across imaging wavelengths, consistent with interference ring behavior.

**Table 2:** Linear regression statistics of dark fringe radius^2^ measurements (dependent) vs. Fringe order on cSLO color fundus photography (# observations = 5 at each *λ* per eye)

Patient	Eye	λ	R2	**Adjusted R**^**2**^	SE	F-statistic (Sig. F)
1	OD	R	0.972	0.963	4.05 ×10^−8^	105.0 (0.002)‡
		G	0.985	0.971	4.02 ×10^−8^	99.7 (0.002)‡
		B	0.973	0.965	3.71 ×10^−8^	109.9 (0.002)‡
	OS	R	0.922	0.896	4.16 ×10^−8^	35.3 (0.0095)†
		G	0.931	0.908	3.22 ×10^−8^	40.6 (0.008)†
		B	0.950	0.934	1.74 ×10^−8^	57.5 (0.0048)‡
2	OD	R	0.976	0.967	5.27 ×10^−8^	119.9 (0.002)‡
		G	0.947	0.929	6.34 ×10^−8^	53.5 (0.005)†
		B	0.937	0.916	5.71 ×10^−8^	57.5 (0.0048)‡
	OS	R	0.911	0.881	1.18 ×10^−7^	30.5 (0.01)*
		G	0.947	0.930	5.86 ×10^−8^	54.1 (0.005)†
		B	0.921	0.895	4.27 ×10^−8^	35.1 (0.0096)†
3	OD	R	0.950	0.933	2.18 ×10^−8^	57.1 (0.005)†
		G	0.933	0.911	2.36 ×10^−8^	41.9 (0.008)‡
	OS	R	0.974	0.965	2.02 ×10^−8^	110.8 (0.002)‡
		G	0.956	0.941	2.45 ×10^−8^	64.6 (0.004)‡
**Average**			**0.949**	**0.932**		
**SD**			**0.022**	**0.023**		
**Median**			**0.949**	**0.932**		
**Range**			**0.911 - 0.985**	**0.881 - 0.971**		

λ = wavelength of light; B = blue (λ = 488 nm); G = green (λ = 532 nm); *R* = red (λ = 635 nm); R^2^ = coefficient of determination; SD = standard deviation; SE = standard error

* *p* < 0.05

† *p* < 0.01

‡ *p* < 0.005

**Table 3:** Linear regression coefficients of dark fringe radius^2^ measurements (Dependent) vs fringe order on cSLO color fundus photography (# observations = 5 at each *λ* per eye)

		λ	Intercept: Coefficient (SE)	X Variable (Dark Fringe Order): Coefficient (SE)
Patient 1	OD	R	-1.53 ×10^−7^ (4.25 ×10^−8^)*	1.31 ×10^−7^ (1.28 ×10^−8^)‡
		G	-1.53 ×10^−7^ (4.22 ×10^−8^)*	1.27 ×10^−7^ (1.27 ×10^−8^)‡
		B	-1.51 ×10^−7^ (3.89 ×10^−8^)*	1.23 ×10^−7^ (1.17 ×10^−8^)‡
	OS	R	-9.51 ×10^−8^ (4.37 ×10^−8^)	7.82 ×10^−8^ (1.32 ×10^−8^)†
		G	-7.92 ×10^−8^ (3.38 ×10^−8^)	6.48 ×10^−8^ (1.02 ×10^−8^)†
		B	-5.19 ×10^−8^ (1.83 ×10^−8^)	4.18 ×10^−8^ (5.51 ×10^−9^)‡
Patient 2	OD	R	−2.25 ×10^−7^ (5.53 ×10^−8^)*	1.83 ×10^−7^ (1.67 ×10^−8^)‡
		G	−1.89 ×10^−7^ (6.65 ×10^−8^)	1.47 ×10^−7^ (2.00 ×10^−8^)†
		B	−1.63 ×10^−7^ (5.99 ×10^−8^)	1.20 ×10^−7^ (1.81 ×10^−8^)†
	OS	R	−2.92 ×10^−7^ (1.24 ×10^−7^)	2.07 ×10^−7^ (3.74 ×10^−8^)*
		G	−1.82 ×10^−7^ (6.15 ×10^−8^)	1.36 ×10^−7^ (1.85 ×10^−8^)†
		B	−9.83 ×10^−8^ (4.48 ×10^−8^)	8.01 ×10^−8^ (1.35 ×10^−8^)†
Patient 3	OD	R	−6.41 ×10^−8^ (2.29 ×10^−8^)	5.22 ×10^−8^ (6.91 ×10^−9^)‡
		G	−6.51 ×10^−8^ (2.47 ×10^−8^)	4.83 ×10^−8^ (7.45 ×10^−9^)†
	OS	R	−8.00 ×10^−8^ (2.12 ×10^−8^)*	6.72 ×10^−8^ (6.38 ×10^−9^)‡
		G	−8.23 ×10^−8^ (2.57 ×10^−8^)*	6.23 ×10^−8^ (7.75 ×10^−9^)‡

λ = wavelength of light; B = blue (λ = 488 nm); G = green (λ = 532 nm); *R* = red (λ = 635 nm); SE = standard error

* *p* < 0.05

† *p* < 0.01

‡ *p* < 0.005

### Wavelength Dependence of Concentric Macular Fringe Patterning

To assess wavelength dependence, experimental and theoretical ratios of dark-fringe radius^2^ were compared at known wavelengths (Tables 4, 5, 6). Mean experimental ratios (r^2^_red_:r^2^_green_, r^2^_green_:r^2^_blue_, and r^2^_red_:r^2^_blue_ were consistently larger than theoretical Newton’s ring predictions (*p* < 0.05). Effect sizes were greatest in comparisons involving shorter wavelengths (Cohen’s d = 0.4, 1.0, and 0.8, respectively). These findings suggest wavelength-dependent deviation from idealized interference conditions, possibly reflecting retinal or optical factors not incorporated in the theoretical model.

**Table 4:** Experimental and theoretical ratios of dark fringe radius^2^ at different wavelengths of light (red = 635 nm; green = 532 nm; blue = 488 nm) for the first five CMR with one-sample T-Test results comparing experimental to theoretical ratios

ID	Eye	Order (n)	r^2^ red: r^2^ green		r^2^ green: r^2^ blue		r^2^ red: r^2^ blue	
			EXP	THE	EXP	THE	EXP	THE
1	OD	1	1.2276	1.1936	1.2069	1.0902	1.4816	1.3012
		2	1.1813		1.0982		1.2973	
		3	1.0691		1.0552		1.1281	
		4	1.0405		1.0156		1.0567	
		5	1.0464		1.0460		1.0945	
	OS	1	1.7220		1.6715		2.8784	
		2	1.1064		2.0268		2.2424	
		3	1.1477		1.5806		1.8141	
		4	1.2414		1.3569		1.6846	
		5	1.2092		1.6569		2.0035	
2	OD	1	1.3588		1.3181		1.7910	
		2	1.0861		1.6017		1.7397	
		3	1.4692		1.2884		1.8930	
		4	1.3904		1.2594		1.7510	
		5	1.1939		1.2282		1.4664	
	OS	1	1.3260		1.1852		1.5715	
		2	1.2696		1.1588		1.4713	
		3	1.0220		1.3198		1.3489	
		4	1.6972		1.9959		3.3875	
		5	1.4434		1.5771		2.2763	
3	OD	1	1.7107		n.a.		n.a.	
		2	1.4397					
		3	1.2857					
		4	1.1302					
		5	1.1006					
	OS	1	2.0739		n.a.		n.a.	
		2	1.4277					
		3	1.2516					
		4	1.1702					
		5	1.0842					
**One-Sample t-Test**			n = 30		n = 20		n = 20	
**Mean (SD)**			1.2974 (0.2454)		1.3824 (0.2980)		1.7689 (0.5843)	
**t-Statistic (df)**			2.317 [15]		4.384 [24]		3.580 [24]	
**p-Value**			0.03*		< 0.001***		0.002***	
**Mean Difference [95% CI]**			0.1038 [0.0122, 0.1955]		0.2922 [0.1527, 0.4316]		0.4678 [0.1942, 0.7412]	
**Cohen’s d,** **[95% CI]**			0.423 [0.046, 0.794]		0.980 [0.435, 1.508]		0.800 [0.287, 1.298]	

CI = confidence interval; CMR = concentric macular rings; EXP = experimental; ID = patient identifier; n.a. = not applicable; SD = standard deviation; THE = theoretical, calculated by λ_1_/λ_2_

* *p* < 0.05

** *p* < 0.01

*** *p* < 0.005

**Table 5:** Difference between experimental and theoretical ratios of dark fringe radius^2^ at different wavelengths of light (red = 635 nm; green = 532 nm; blue = 488 nm) for the first five CMR of all patients

ID	Eye	Order (n)	DIFF r^2^ red: r^2^ green (%)	DIFF r^2^ green: r^2^ blue (%)	DIFF r^2^ red: r^2^ blue (%)
1	OD	1	2.8	10.7	13.9
		2	−1.0	0.7	−0.3
		3	−10.4	−3.2	−13.3
		4	−12.8	−6.8	−18.8
		5	−12.3	−4.1	−15.9
	OS	1	44.3	53.3	121.2
		2	−7.3	85.9	72.3
		3	−3.8	45.0	39.4
		4	4.0	24.5	29.5
		5	1.3	52.0	54.0
2	OD	1	13.8	20.9	37.6
		2	−9.0	46.9	33.7
		3	23.1	18.2	45.5
		4	16.5	15.5	34.6
		5	0.0	12.7	12.7
	OS	1	11.1	8.7	20.8
		2	6.4	6.3	13.1
		3	−14.4	21.1	3.7
		4	42.2	83.1	160.3
		5	20.9	44.7	74.9
3	OD	1	43.3	n.a.	n.a.
		2	20.6		
		3	7.7		
		4	−5.3		
		5	−7.8		
	OS	1	73.7	n.a.	n.a.
		2	19.6		
		3	4.9		
		4	−2.0		
		5	−9.2		
**% DIFF**		**Average (SD)**	**8.7 (20.6)**	**26.8 (27.3)**	**21.7 (32.8)**
		**Median (Min, Max)**	**3.4 (−14.37, 73.7)**	**19.5 (−6.8, 85.9)**	**13.5 (−18.17, 160.3)**

CMR = concentric macular rings; DIFF = difference between experimental and theoretical ratios [(theoretical r^2^ – experimental r^2^)/theoretical r^2^] x 100%); ID = patient identifier; n.a. = not applicable; SD = standard deviation

**Table 6:** Paired T-tests comparing % absolute difference between experimental and theoretical ratios of dark fringe radius^2^ at different wavelengths of light in CMR of patients 1 and 2

Descriptive Statistics			Paired t-Test Results			
ABS DIFF r^2^ ratio	Average (SD)	Median (Min, Max)	Comparison Groups	Mean Difference [95% CI]	t-Statistic (df), p	Cohen’s *d*, [95% CI]
R:G (%), *n* = 20	12.9 (12.2)	10.8 (0.0, 44.3)	**R:G vs G:B**	−15.3 [−26.3, −4.4]	−2.9 [24], **p = 0.009†**	−0.655 [−1.132, −0.163]
G:B (%), *n* = 20	28.2 (25.8)	19.5 (0.7, 85.9)	**G:B vs R:B**	−12.6 [−23.8, −1.3]	−2.3 [24], p = 0.03*	−0.521 [−0.983, −0.047]
R:B (%), *n* = 20	40.8 (40.3)	31.5 (0.3, 160.3)	**R:B vs R:G**	27.9 [13.2, 42.6]	4.0 [24] **p < 0.001‡**	0.888 [0.359, 1.400]

ABS DIFF = Percent Absolute Difference of r^2^ ratios at different wavelengths of light, calculated by [(absolute value (theoretical r^2^ – experimental r^2^)/theoretical r^2^] x 100%); B = blue (488 nm); CI = confidence interval; CMR = concentric macular rings; *r* = radius of dark fringe; *R* = red (635 nm); G = green (532 nm); SD = standard deviation

* *p* < 0.05

† *p* < 0.01

‡ *p* < 0.005

### Henle fiber layer corrugations on optical coherence tomography

**Fig. 5:**
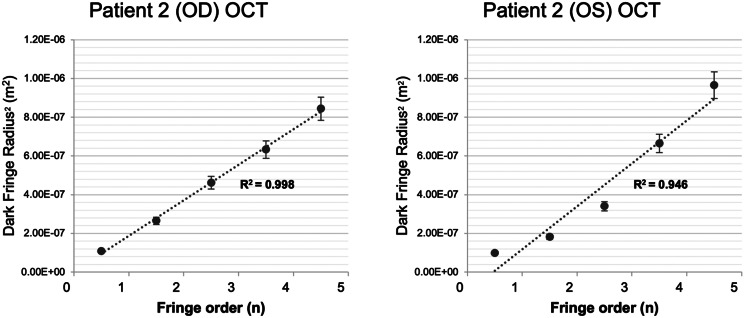
Dark fringe radius^2^ (m^2^) compared to fringe order of outer plexiform layer areas of hyporeflectivity on spectralis optical coherence tomography (OCT) of patient 2 OU. Linear curve fit to measurements (*n* = 5) at wavelength 850 nm ±60 nm. Linear regression was performed with R2 values of 0.998 and 0.946 OD and OS, respectively. Error bars include relative uncertainty (0.0706*r^2^) and absolute uncertainty (25 × 10^−10^ m^2^)

OCT imaging revealed alternating hyper- and hyporeflective bands within the OPL/HFL, corresponding to the en face CMR patterns seen on cSLO fundus imaging (Figs. 1, 3H–I). Linear regression of dark-fringe radius^2^ versus fringe order demonstrated strong linearity in both eyes of Patient 2 (R^2^ = 0.998 OD; 0.946 OS) (Fig. 5, Table 7). Fringe radii on OCT were larger than those on cSLO imaging, consistent with the model prediction that fringe radius increases with wavelength. Together, these results support the interpretation that both cSLO CMR and HFL corrugations reflect interference ring phenomena rather than structural retinal features.

**Table 7:** Linear regression results of dark fringe radius^2^ (dependent) on fringe order of CMR on OCT (λ = 850 nm ±60 nm) of patient 2 OU

Eye	Regression Statistics			ANOVA	Coefficients	
	R2	Adjusted R^2^	SE	F-statistic (Sig. F)	Intercept: Coefficient (SE), p Value	X Variable (Dark Fringe Order): Coefficient (SE), p Value
OD	0.998	0.997	1.579 ×10^−8^	1355.900 (4.40 ×10^−5^)	2.865 ×10−9 (4.992 ×10−8), *p* = 0.9	1.838 ×10−7 (4.992 ×10−9), *p* < 0.001†
OS	0.946	0.928	9.668 ×10^−8^	52.442 (5.43 ×10^−8^)	−1.033 ×10−9 (8.782 ×10−8), *p* = 0.3	2.214 ×10−7 (3.057 ×10−8), *p* = 0.005*

λ = wavelength of light; CMR = concentric macular rings; R^2^ = coefficient of determination; SE = standard error

* *p* < 0.01

† *p* < 0.005

## Discussion

### Summary of findings

Through modality-agnostic physics-based analyses, our findings suggest that the morphology of CMR and HFL corrugations in FH is most consistent with coherent optical interference, supporting an imaging-artifact origin rather than intrinsic retinal structure. Across all cSLO and OCT, squared fringe radius (*r*^*2*^) scaled linearly with fringe order (*n*) and increased with wavelength (*λ*), hallmark signatures of Newton-like interference that fixed anatomy, with wavelength-invariant geometry, cannot produce (Figs. 4–5, Table 4).

### Interpretation of concentric macular rings in foveal hypoplasia

First described in the English literature by Cornish, Reddy, and McBain in 2014, CMR in FH were believed to be due to polarization of the radially symmetric RNFL and HFL [2]. An anatomic origin was inferred from multimodal presence [2]. Lee, Woertz, et al. proposed cellular-level differences (Müller glia or cone axon size) [26]. Subsequent work invoked anatomy: Ramtohul, Comet, and Denis postulated that the absence of centripetal HFL displacement results in vertically oriented Henle fibers, producing CMR, and related CMR to abnormal HFL reflectivity on OCT [4]. Bringmann, Barth, and Ziemssen reported HFL thickness differences and reflectivity changes, attributing these to cystoid spaces and Müller cell gliosis.[5].

Our results offer an alternative interpretation: the observed r^2^-order linearity and wavelength scaling of CMR geometry are characteristic of interference. While recent suggestions that CMR are artifacts are confined to abstracts or case images, our review uncovered a largely uncited 1995 German-language report in which Wodowosow and Swerdlin documented CMR using chromato-ophthalmoscopic examination in 19 patients with albinism and proposed a Newton’s-rings interference origin, without quantitative analysis.[9].

### The formation of concentric macular ring interference artifacts

In coherence-based retinal imaging (cSLO and OCT), ring-like macular patterns can arise from optical interference rather than anatomy: when two partial retinal reflections with nearly equal optical path lengths reach the detector, their phases add or cancel to produce alternating light and dark rings [17, 18, 27, 28]. Ring visibility depends on coherence: under white-light ophthalmoscopy, low temporal coherence and spectral averaging reduce contrast, so rings are typically absent even when they appear in cSLO and OCT [19, 20, 27]. Concentric patterns indicate a smoothly varying optical path difference across the macula; a radially varying path naturally yields circular fringes without requiring a specific microstructure [17, 18, 27, 28]. Interference phenomena are widely used in ophthalmology, including lens quality assessment and the interferometric formation of OCT images.[18, 21].

Interference ring visibility and morphology depend on imaging geometry. Patterns are strongest at near-normal incidence and are attenuated by surface irregularity [15, 17, 27]. Departures from radial symmetry yield ovoid or attenuated rings, consistent with Patient 2 OS (wider horizontal CMR) and astigmatic foveal curvature on OCT (Fig. 1C,F,G).[15, 17, 27].

**Fig. 6:**
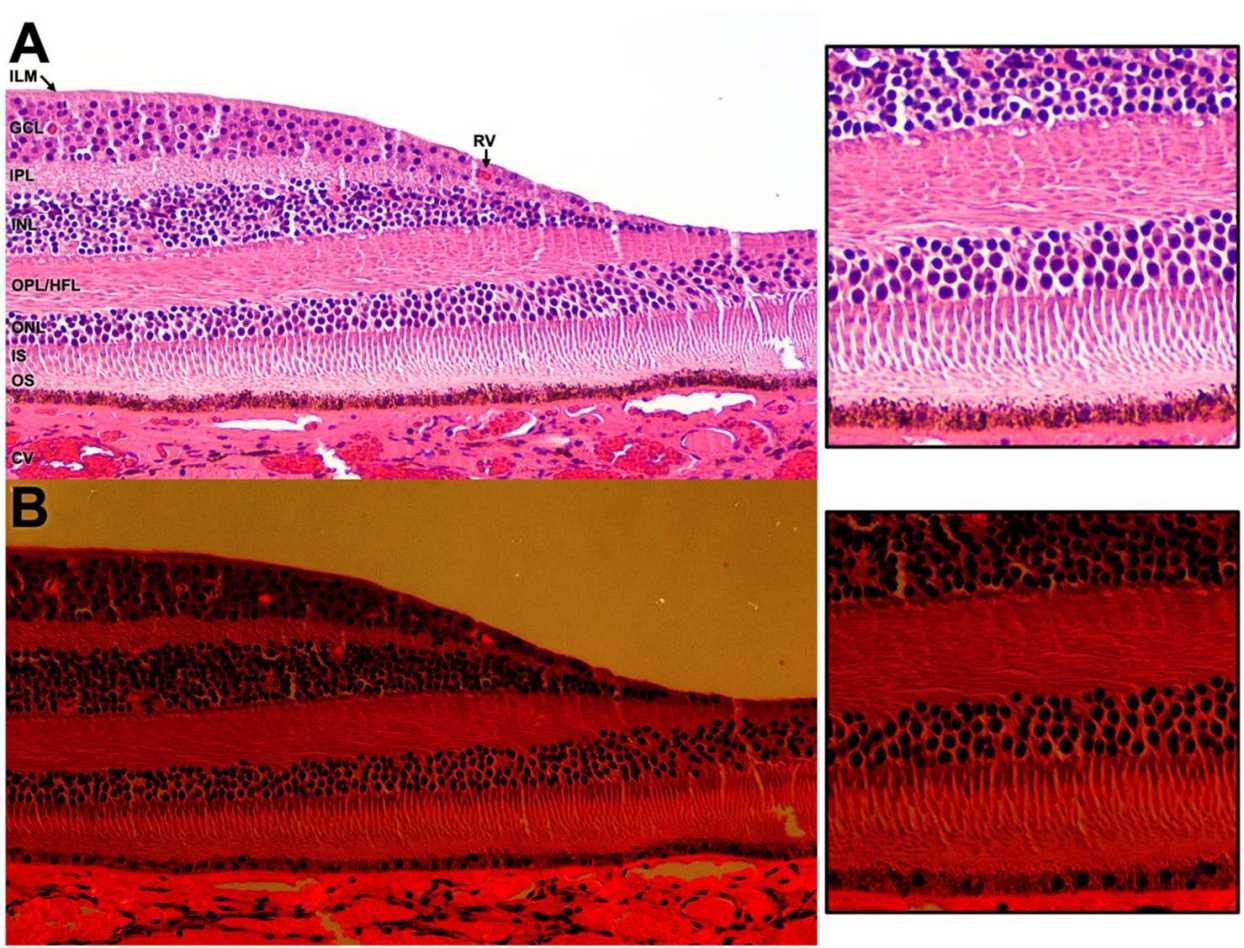
Normal fovea; glutaraldehyde-fixed and paraffin-embedded, stained with hematoxylin-eosin, white light photomicrograph. **A**. Non-polarized image demonstrating radial orientation of the Henle’s fiber layer (hfl). Inset highlights outer retina detail. ilm – internal limiting membrane, gcl – ganglion cell layer, rv – retinal vessels, ipl – inner plexiform layer, inl – inner nuclear layer, OPL/HFL – outer plexiform layer/Henle’s fiber layer, onl – outer nuclear layer, is – inner segment of photoreceptors, os – outer segment of photoreceptors, CV – choroidal vessels. **B**. Polarized white light photomicrograph shows eosin-enhanced polarization within retinal and choroidal tissue, most prominent at the level of the ilm, ipl, OPL/HFL, os, rv, and CV. Inset highlights polarization within the HFL and OS. [**A**,**B**: 200x, insets–600x]

**Fig. 7:**
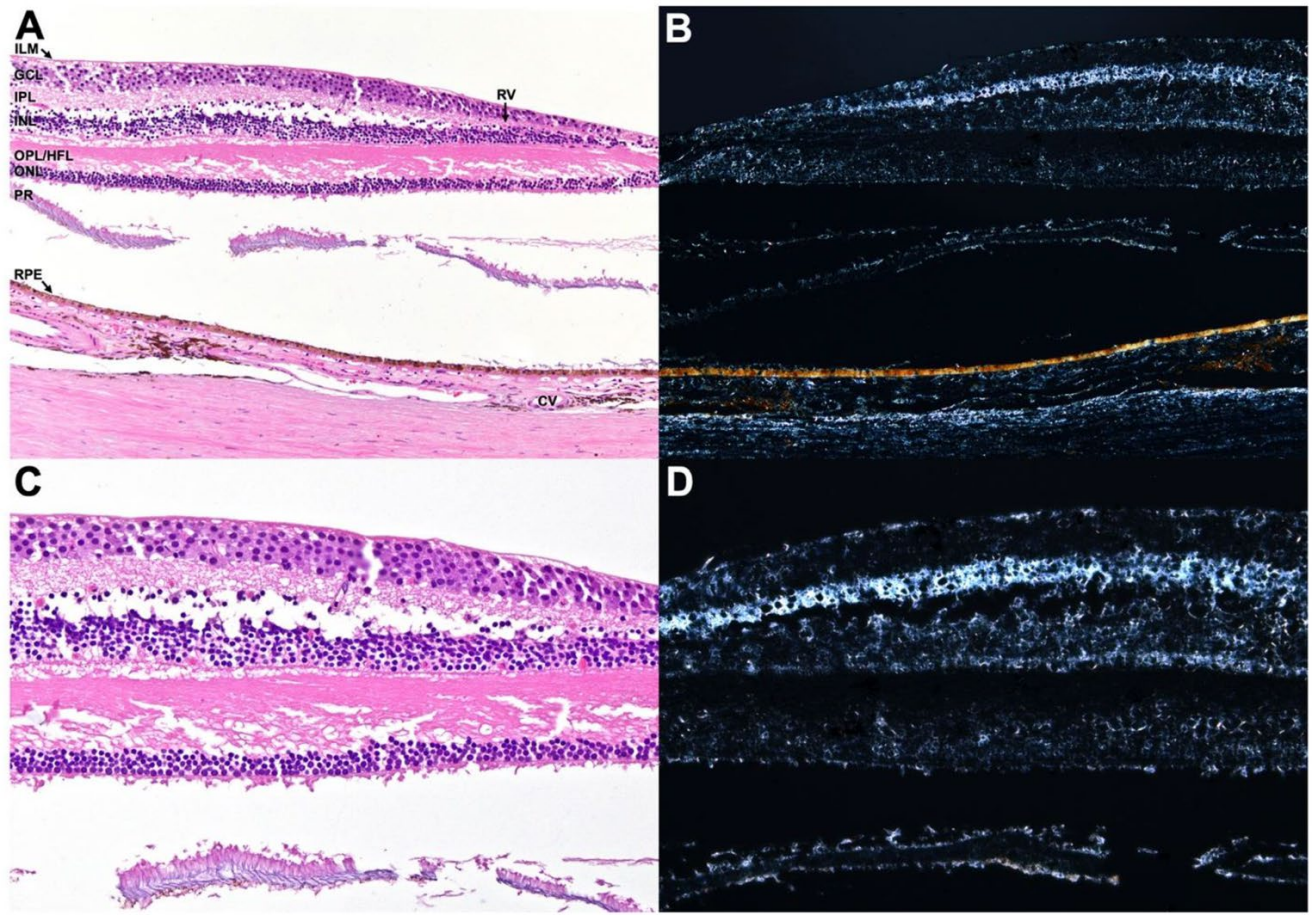
Normal fovea, with artifactitious detachment of photoreceptors (PR) from outer nuclear layer (ONL) and retinal pigment epithelium (RPE); formalin-fixed and paraffin-embedded, white light photomicrograph. **A**. Non-polarized image of hematoxylin and eosin (H&E) stained slide showing retinal architecture. ILM – internal limiting membrane, GCL – ganglion cell layer, IPL – inner plexiform layer, RV – retinal vessels, INL – inner nuclear layer, OPL/HFL – outer plexiform layer/Henle’s fiber layer, CV – choroidal vessels. **B**. Polarized image of unstained slide showing polarization of retinal and choroidal tissue, most prominent in the IPL, RPE, and choroical stroma and also observed in the ILM, RV, INL, OPL/HFL, PR, and CV. **C**. Non-polarized image of hematoxylin and eosin (H&E) stained slide highlighting retinal detail. **D**. Polarized image of unstained slide showing polarization of the ILM, RV, IPL, INL, OPL/HFL, and PR. A large difference in relative polarization is noted at the interface of the GCL with the IPL and the INL with the OPL/HFL. [A,B: 100x, C,D: 200x]

The fovea exhibits optical anisotropy (birefringence) that can modulate optical path and fringe visibility [29–32]. Figures 6–7 show polarization contrast in paraffin-embedded foveal tissue, with a pronounced band near the OPL/HFL-inner nuclear layer (INL) junction corresponding to the synaptic junction between photoreceptor pedicles and INL cells [33]. Prior reports have proposed different interfaces for ring formation (e.g., ILM, RPE, HFL) [8]. The present study does not identify a specific layer pair or exact optical pathway; it establishes only that the observed ring geometry follows interference behavior across modalities.

### Concentric macular ring interference artifacts beyond foveal hypoplasia

Because optical interference is nonspecific, CMR are not unique to foveal hypoplasia and have been reported across congenital, genetic, and acquired retinal disorders [34–42]. Subtle “fingerprint-like” patterns and HFL corrugations also occur in some normal eyes and are typically less organized and attenuated than in disease [42]. Variations in the contour and orientation of retinal layers may impose a radially varying optical path that generates concentric interference fringes. This unified framework cautions against interpreting CMR as a disease-specific hallmark and provides a common mechanism across diverse conditions.

## Limitations

This proof-of-concept study is limited by its small sample size and retrospective design. Only three patients (six eyes) with FH were included, all with *OCA2*-associated albinism; thus, findings may not generalize to other genetic or acquired causes of FH. Measurements were obtained by a single grader, and although repeated for consistency, inter-grader reproducibility was not assessed. Also, only the first five dark fringes were analyzed, and higher fringe orders could reveal additional optical influences. Despite these constraints, the clear adherence of CMR and HFL corrugations to the physical relationships of interference across multiple wavelengths and imaging modalities strongly supports the validity of the proposed mechanism. Future multicenter studies including larger and genetically diverse cohorts, reproducibility assessments, and complementary optical modeling will be valuable to further test and refine this hypothesis.

## Conclusions

Across cSLO and OCT, CMR geometry showed linear r^2^-order dependence and wavelength scaling—hallmarks of coherent interference that fixed anatomy cannot reproduce. These findings support that CMR and HFL corrugations in FH are extrinsic optical interference artifacts, rather than intrinsic retinal structures. While such patterns may still serve as clinical indicators of altered retinal architecture, they are nonspecific to FH. Accordingly, caution is necessary when interpreting these imaging features, which may represent artifactitious optical phenomena rather than true cellular anatomy.

## Data Availability

All data generated or analysed during this study are included in this published article and its supplementary information files.

## Abbreviations

R^2^: Coefficients of determination
CMR: Oncentric macular rings
HFL: Henle fiber layer
OCT: Optical coherence tomography
n: Fringe order
λ: Wavelength
r^2^: Radius^2^
FH: Foveal hypoplasia
FAZ: Foveal avascular zone
RPE: Retinal pigment epithelium
RNFL: Retinal nerve fiber layer
cSLO: confocal scanning laser ophthalmoscopy
NIRR: Near-infrared reflectance
RGB: True color fundus photo
RG: Two color fundus photo
FAF: Fundus autofluorescence
INL: The inner nuclear layer
OPL: Outer plexiform layer
OU: In both eyes
CHRRPE: Combined hamartoma of the retina and retinal pigment epithelium
VKH: Vogt-Koyanagi-Harada disease
ERM: Epiretinal membrane
RRD: Rhegmatogenous retinal detachment
H&E: Hematoxylin and eosin
